# Short Notes on Some of the Details of Sanitary Police

**Published:** 1855

**Authors:** Robert Druitt

**Affiliations:** Licentiate of the Royal College of Physicians, London.


### SHORT NOTES ON SOME OF THE DETAILS OF SANITARY POLICE.

By Robert Druitt, Licentiate of the Royal College of Physicians, London.

### No. I.—ON THE DAILY REGISTRATION OF FACTS BY MEDICAL PRACTITIONERS.

Great Truths, we are assured by good authority, often lie hid at the roots of words; and many falsehoods and prejudices might be got rid of by a course of etymology. The word *police* is a not bad illustration of this position. Taken in its true and original meaning, it signifies the art or science of the government of cities, embracing all that is necessary in order that human beings, instead of leading roving, precarious, isolated lives, may be organized into communities, and dwell in fixed and permanent habitations, for the prosecution of those studies and occupations which cannot be carried on without association. In this sense, the science of *police* becomes a liberal and ennobling study. In its vulgar acceptation it is nothing more than the machinery for thief-catching, and conveys no ideas that are not repulsive and degrading.

Curious enough it is to notice how entirely the duties of a *police force* are confined, in the vulgar idea, to one or two functions, such as the preservation of the public peace, and the prevention or punishment of violence against persons, and depredations of property. Yet surely where multitudes of human beings are brought together, there are numbers of circumstances arising out of this very fact, and requiring deliberate provision and control; in other words, there are branches of police infinitely higher and more important than that which relates merely to crime. Both in the moral and physical world, every miasm acquires tenfold virulence by concentration. One vicious person, or one source of unwholesome vapour, may be harmless. It is in crowds that infection of all kinds acquires the power and opportunity of spreading. It cannot therefore be a perfect system of police, under which masses of a town population can be allowed to grow up in ignorance, idleness, and vice, ready to prey like vermin on the industrious and frugal. It is equally true that a real system of police must provide for the various circumstances, inseparable from the aggregation of human beings within narrow limits, by which health may be affected. Yet what a revolution must take place in the vulgar mind before health can be considered within the province of a police force, or before those who are prosecuted for nuisance will be looked on as aught than martyrs in the ancient cause of British liberty and dirt.

If we may parody the words of the immortal W. S.,—

"——who steals my purse,"

(which very parody, by the way, ought in a well-regulated community, as I humbly confess, to be punished); "he who filches from me my good *health*, doth make me poor, indeed." As it is, however, there is a police who take up the filcher of sixpences and send him to Bridewell; whilst the man who robs the poor of their health by letting poisonous lodgings, which they have no choice but to occupy—this man is respectable, drives to his country seat, and possibly is a magistrate.

I trust the reader will pardon me for my zeal in rescuing a good word from a state of degradation, and in maintaining under cover of an etymological argument that the health and morals of a community are as legitimate objects of government as its property; and that there ought to be functionaries to inquire into offences against health, with ample powers (for that is the thing needful) to enforce measures necessary for the preservation of the community against fever, a foe more subtle and destructive than the perpetrators of violence or fraud. Having relieved my mind by this burst of etymology, I beg permission to offer a few jottings on some of the details which would come under the cognisance of a well constituted sanitary police force; such as the state of the surface of roads, streets, and pavements; the arrangement of streets with respect to the proper supply of light and air, and the construction of efficient sewers; the purity of the atmosphere, and so forth. I will begin with a few remarks upon that which is the very life and soul of all science, and which is more particularly needful in the infant science of sanitary police—I mean the collection of facts. And I wish, at present, to dwell upon the advantages which would ensue, if medical practitioners of all ranks and specialties were to keep daily record of the cases which come under their observation.

This was long since urged upon the profession by Mr. Farr in the "Circular addressed to Medical Practitioners and to Registrars, with the Statistical Nosology," &c., issued from the Registrar-General's Office in 1845 ; and I beg permission to quote the suggestions in question, and the mode in which they were advised to be carried into effect.

"NOTE.—In order to be in a position to return the causes of death accurately, medical practitioners are earnestly recommended to keep brief memoranda of all the cases which they attend, in the simplest form, such as is given below. The private register of the medical practitioner would at the end of a few years be of incalculable interest to him: he might refer back to it for important information, transmit it to his sons or successors in practice, analyse the results of his experience; and, in conjunction with his brethren all over the country, would ultimately accumulate a vast series of materials, which could not fail to advance medical science.

"Engaged in the practice of a laborious profession, medical men should not attempt too much at first; all cases should be noticed; but those facts should be chiefly recorded which are of an unequivocal nature, and that admit of precise statement and comparison in respect to number, time, weight, and measure.

| No. | Name of Patient and Residence. | Sex. | Age last Birth-day. | Profession; Habits. | Diseases. | | Dates of Important Facts in the Case; and of the resulting Recovery or Death. |
|---|---|---|---|---|---|---|---|
| | | | | | Primary and Secondary. | Date of their Appearance. | |
| 1* | John Jones 7, Drury-lane | M. | 7 | A day scholar; (father a master tailor). | Measles; Pneumonia. | Mar. 5. Mar. 12. | Patient first seen Mar. 9; the eruption of measles then visible; appeared on the 8th; cough, rust-coloured sputa, crepitation, 12th; left lung consolidated, 14th; last seen Mar. 19th; d. Mar. 20th. Post mortem: left lung hepatised; pleura adherent. |

* "The number will refer to the Prescription-book for treatment."

Very excellent this scheme would be if put into execution. But I am sufficiently acquainted with the habits of the medical practitioner to know that it presents some difficulties.

The first difficulty in the case is, that the making memoranda of cases is, to those who have never been fortunate enough to be trained to it, most irksome and fatiguing; and the being obliged to take down, open, and make entries in, a *separate* book, is a serious effort to the wearied and harassed mind of our country friends.

In the next place, the formality of the eight columns, and the allotment of the same space to all cases, are obstacles. And besides, a summary of facts drawn up at the end of a case, although very valuable, is yet far less valuable than a much less formal series of notes would be, if jotted down on the spur of the moment; in fact, it ought to be a compilation and condensation of such notes.

I would therefore propose a plan by which no fresh book should be required, and by means of which every entry should be made as early as possible after the occurrence of the fact which it records.

I need scarcely say that medical practitioners do keep records, and in large practices very voluminous ones; but in ninety cases out of a hundred, these are available only as records of treatment, and as means by which the patient's pecuniary liabilities may be calculated. For instance, a general practitioner, who receives payment for his attendance and for the medicine which he supplies, must keep two books, the day-book and the ledger. In the first he enters each day's work in the following form, there being one column for the name and address, and another for the entering in abbreviated Latin, of treatment or attendance:—

| | |
|---|---|
| Johnson, Sir Theophilus, Bart.<br>17, Queen Street. | Visit: noct: |
| | Appl: Hirud: × lateri sinistro |
| | ℞ Hydrarg: Chlorid: gr. vj<br>Pulv: Opii gr. ii<br>Ft pil: statim |
| | ℞ Magnes: Sulph: ʒiii<br>Inf. Sennæ c: ℥xiii<br>Ft. haust: post hor: 2 s. |

And so on for each patient in succession. In the ledger, there is of course a page devoted to each separate person, in which all the attendance and medicines supplied to such person during a given time are summed up, so that he may be furnished with his account.

The number of volumes of day-books, filled with details of visits, draughts, mixtures, and all the other delectable varieties of medicines, that accumulate after years of extensive practice, are immense, but for all scientific purposes are valueless. Members of my own family have practised the medical profession for nearly two centuries in Dorsetshire; and I have looked through many day-books in the hope of discovering some data that would throw light on the question as to the alleged change in disease from an inflammatory to a low type, during the last twenty or thirty years, but my search has been fruitless.*

That which I propose, in order to convert these dead books into useful, curious, and valuable documents, is very simple; perhaps my readers may think it contemptible; but never mind; I am fully convinced that it is only some very simple plan that *can* or will be adopted by the hardworked members of the profession. It is this:—Add a narrow column to the day-book, so that, alongside the prescription, the practitioner may jot down any fact worth remembering as to the patient's symptoms or condition. No matter how short these jottings may be; if they are authentic, done on the spur of the moment, when the facts are fresh in the mind, they will be in time of immense value.

My scheme requires no additional book and no *red-tapery.* It may be worked off-hand, and is not a separate task, as the making entries in a fresh book would be. The only thing

* I possess a book, containing entries of treatment of patients in 1689, together with a list of the drugs used by country apothecaries, and their prices; copies of prescriptions by eminent physicians of the day, such as Glisson, Highmore, and Sydenham. In particular, there is a "Masse of Pills prescrib'd for Mrs. Gibbon, by Dr. William Highmore," containing twenty-six ingredients. One of my ancestors (whose Christian name, Sacheverell, bespeaks the Tory opinions of his family and the time of his birth), began practice in 1742, and made a few entries of the disorders for which he treated his patients. Thus, a Mr. Barns, was affected by "Epidemicall Feavour," August 14, 1742, and treated by bleeding to ten ounces, and various cordials and opiates. A Mr. Biddle, of Cogden, who had a "Great Chill, attended by a Feavour, and eruption on the skin;" and Mr. Eldridge's wife, who had "a Cold settled at the Pit of y^e Stomach," were likewise treated by bleeding, antimony, and opiates. In fact, there seem to have been but few patients at this date, who were not bled. It would be out of place to enter further into particulars of mere antiquarian interest. I will only notice the homely but kind-hearted old English feeling displayed in the titles of patients; for instance, *Goodman* Vivian (who had strangury in 1689), *Goody* Ford, and *Gaffer* Short: *Gaffer = Gevatter, compère, gossip.* All these good names are now merged in the insipid *Mister*, or the ridiculous *Esquire.*

required to make it perfect is a full alphabetical index containing the name of every patient, with reference to the page in the ledger, in which each name appears. The entries in the ledger will give the proper reference to the day-book.

I need not enlarge upon the comfort which such a daily record would afford to the practitioner himself; the advantage of being able to refer to a notice of former symptoms, in order to guide him in his diagnosis; the confidence his patients would place in him, knowing that he possessed a written record of their maladies, and of course that he must *know their constitutions;* and, in particular, the power, in case of a very long standing or disputed account, of verifying each doubtful item;—my present purpose is to shew the gain that would accrue to professional knowledge in general, and to the science of sanitary police in particular.

With regard to epidemics and to cholera, as an example, there are many questions that might be set at rest as to their origin and mode of spreading. Does the cholera depend on a specific poison, derived from the bodies of the sick, and swallowed, as is alleged by one eminent physician, in the water or in some other vehicle by those who are the subjects of the disease? Whether this be true or not, is there any general impression on the health of an entire population within certain limits of space? For example, is it true that, during the prevalence of cholera, the intestinal functions of the whole population are in a state of exalted activity? Is it true that a moderate dose of an ordinary purgative, which at other times would produce very slight effect, is liable, as a rule, to occasion serious overpurging during the prevalence of cholera?

These questions might soon receive positive answers, if a dozen or two of practitioners could furnish any memoranda of actual facts on one side or the other. It is most probable that the evidence which has presented itself to any one individual has been sufficient to give him what is commonly called an *impression.* And the greater part of the profession is guided in almost all things by *impressions,* arising from a few well marked instances. But that which is an impression in the mind of one man becomes strong probability if created in many minds simultaneously, and may be developed into an established rule for practice if found to be generally diffused.

Now we know very well that there are in this town hundreds of individuals who resort to mild aperient medicines, chiefly of the aloetic sort, very frequently. Is it, then, true or not, that the use of such medicines during the months of August and September last was diminished? or that patients complained of the severity of their effects? My private *impression* is in the affirmative.

This is but a partial example. There are other questions of a more general nature, which might receive solution; such as the amount of illness in the entire population, in every rank and at every age, in every season and locality—questions which are of immense importance, and which cannot be solved by returns of mortality merely. Regarding epidemic diseases, it is most necessary that the times of their appearance, the streets and houses infected, should be accurately known by the Minister of Public Health; and not only so, but it is desirable to convert into ascertained *certainty* or otherwise, the *impression* which generally, and apparently with justice, prevails—that the same causes which in great intensity produce well-marked and violent diseases of the so-called zymotic class, may in less intensity produce minor anomalous maladies. For example, I know a house inhabited by a large number of females, milliners by occupation; the house is ill drained, and the lease too short to render it desirable for the tenant (so she thinks) to repair. In July 1852, there was one case of severe small-pox, in a vaccinated adult; in August, another, less severe; at the same time, one or two inmates suffered from precursory feverish symptoms; and during the next six months there were never wanting cases of anomalous papular eruptions.

In a remarkably well drained and healthy house occupied by a tradesman, his son, a youth of seventeen, had well-marked typhoid fever, in August, 1853, and recovered. A maid-servant next month had the same malady, and recovered likewise. At Christmas, the daughter, a fine girl of twenty-one, was seized with it, and died after a week's illness. But in the course of the autumn, the mother and an aunt suffered, as I believe, from the same malady, in a mitigated and anomalous form, though neither of them kept bed, or submitted to medical treatment. The aunt went about the house for many days, shivering, weak, and miserable; she lost flesh, and was disordered in the bowels, but actually attended to the shop all the time. She soon afterwards became deaf, and lost her hair; both of which symptoms had appeared in the case of the youth first affected. The mother soon afterwards began to experience the same symptoms; but after she had been ill a week, seeing her accidentally in the shop, I advised her to put herself into a railway carriage and leave town. She did so, went to a friend's house, and struggled against her illness without confinement to bed; but she lost her hair, and became deaf likewise. A medical gentleman, who happened to be attending in the family, seeing her casually, said he could not make out what was the matter with her, but she looked as if she had had a fever. The patient refused assistance, however, and returned to town pretty well in two months.

I give this as an illustration of the fact, that debility, dyspepsia, loss of hair, anomalous eruptions, and the like, may in some cases be the effects of small doses of fever miasm. But to obtain certainty in this, we want a knowledge of the places and times at which, and the persons in whom such symptoms prevail, side by side with the records of the existence of the graver and better recognised forms of disease.

Of course I am proceeding on the supposition that the materials which the practitioner may daily collect in this way, for his own comfort and advantage, ought to form the basis of an annual return to the authorities appointed to superintend the public health. I am perfectly certain that such a return as I contemplate, or such a return as the Board of Health requested practitioners to forward to it of the results of the late cholera epidemic, would be much more easily and accurately made, if, instead of being entered from day to day into the Government sheet—a thing which busy men actually could not do—they were jotted down side by side with the prescription for each case in the day-book, and then compiled when the epidemic had ceased, and a more leisurely time had arrived.

But are medical men to do all this for nothing? My limits do not allow me to discuss this point now. I will merely conclude, by urging the stationers who supply medical day-books, to provide them with an extra column for the purpose I am advocating. If such books could be published cheaply, under Government patronage, they would, I believe, be more likely to be put into use.

